# A Case of Typical Hemolytic Uremic Syndrome in an Adult

**DOI:** 10.7759/cureus.3289

**Published:** 2018-09-11

**Authors:** Ramez Kouzy, Rasha Alawieh, Fares Sukhon, Sally Temraz

**Affiliations:** 1 School of Medicine, American University of Beirut Medical Center, Beirut , LBN; 2 Internal medicine, American University of Beirut Medical Center, Beirut, LBN; 3 Hematology & Oncology, American University of Beirut Medical, Beirut, LBN

**Keywords:** hemolytic uremic syndrome, hus, hemolytic anemia, shiga toxin-producing escherichia coli, acute kidney injury, escherichia coli (e. coli), anemia

## Abstract

Typical hemolytic uremic syndrome (HUS) in adults is an uncommon clinical occurrence and has been rarely reported in the literature. Typical HUS is mainly caused by Shiga toxin-producing *Escherichia coli* (STEC) and is typically a pediatric disease. Worldwide outbreaks have been reported, one of the largest and most recent being in Germany. We are reporting a case of a 55-year-old male who presented with acute diarrhea. His laboratory parameters were suggestive of HUS and molecular testing was positive for STEC in stools. The patient received supportive therapy, and he recovered clinically with an improvement in his laboratory parameters. We hereby highlight the importance of timely diagnosis of typical HUS in guiding management and avoiding unnecessary testing and treatment. The mainstay of therapy is aggressive and prompt intravenous hydration to help alleviate the acute kidney injury and improve the clinical outcomes.

## Introduction

Hemolytic uremic syndrome (HUS) belongs to a wide variety of diseases called thrombotic microangiopathies, which primarily refers to pathologies due to vascular damage [1].

Hemolytic uremic syndrome in children has been widely recognized to be caused by Shiga toxin-producing *Escherichia coli* (STEC) such as *E. coli* O157:H7; however, cases of STEC-HUS are rarely reported in adults worldwide [2]. A large outbreak of HUS associated with STEC O104:H4 occurred in Germany in 2011 and affected adults, but overall HUS cases remain concentrated in the pediatric age group [3].

In this case report, we discuss the case of a healthy 55-year-old man presenting with STEC-HUS, predominantly a pediatric disease. We highlight the importance of recognizing HUS from other hemolytic microangiopathies in adults residing in developing nations in order to facilitate early diagnosis and develop a prompt treatment plan.

## Case presentation

A previously healthy 55-year-old man presented to the emergency department with an eight-day history of diarrhea. He reported that his diarrhea started shortly after eating a salad at a restaurant. He described his bowel movements as watery, nonbloody, and nonmucoid in appearance. The patient also complained of generalized fatigue, two episodes of vomiting, nausea, persistent low-grade fever, in addition to jaundice and dark urine. He reported that all his symptoms were acute, and denied any recent travel, hospitalizations, recent antibiotic intake, or unintentional weight loss.

On admission, he had a low-grade fever and was hemodynamically stable. Physical exam was relevant for pallor, jaundice, and mild diffuse abdominal tenderness. Workup revealed a hemoglobin level of 9.8 g/dL, a platelet count of 100,000 /cu.mm, a creatinine level of 3.5 mg/dL, an elevated blood urea nitrogen (BUN) level of 71 mg/dL, a direct bilirubin level of 2.2 mg/dL, a high lactate dehydrogenase (LDH) level of 879 IU/L (normal: 110-265 IU/L), a decreased haptoglobin and normal liver enzymes, prothrombin time (PT), partial thromboplastin time (PTT), and D-dimer levels. On peripheral blood smear, the patient was found to have a moderate number of schistocytes and helmet cells. The ADAM metallopeptidase with thrombospondin type 1 motif 13 (ADAMTS-13) activity and clusters of differentiation (CD) markers specific for paroxysmal nocturnal hemoglobinuria turned out negative. Stool studies were taken and qualitative multiplexed polymerase chain reaction (PCR) for a wide variety of bacteria, parasites, and viruses was done. Shiga-like toxin-producing *E. coli* (STEC) stx1/stx2 was detected in the stool specimen and led to the diagnosis.

During admission, the patient was adequately hydrated with intravenous (IV) fluids and daily laboratory parameters were monitored. On the second day of admission, the fever and diarrhea resolved completely. The patient’s hemoglobin level continued to drop for which he received one unit of packed red blood cells on admission day four. Clinically, the patient markedly improved and that was accompanied by improvement of all his laboratory parameters and he was discharged home on admission day five with scheduled follow up on an outpatient basis.

## Discussion

Hemolytic uremic syndrome is a microvascular occlusive disease characterized clinically by the classical triad of thrombocytopenia, nonimmune microangiopathic hemolytic anemia, and acute renal failure [4-5].

Generally, HUS is divided into two different subsets: classical or typical HUS and atypical HUS (aHUS). Typical HUS is mainly associated with two microorganisms:STEC or less frequently *Streptococcus pneumoniae* [6]. Unlike the typical HUS, aHUS is not usually associated with STEC; on the contrary, the underlying cause has been suggested to be related to the activation and dysregulation of the complement system [7].

Around 5%-15% of patients with STEC develop HUS. In 1983, the first outbreak of STEC O157:H7 related to undercooked beef was reported [8]. To note, STEC-HUS epidemiology is changing and includes, among others, the hybrid STEC O104:H4 in the German outbreak in 2011 which was later traced to fenugreek sprouts [3]. In our case, there was no evidence of an outbreak, and this is in accordance with most of the HUS cases which tend to be sporadic. The known risk factors include undercooked beef and dairy products, contaminated water, animal contact, and human-human transmission. It is important to determine the causative agent of HUS while ruling out aHUS due to significant implications on therapy and prognosis. However, diagnosis can be challenging and requires a high index of suspicion in order to attain a stool sample or a rectal swab promptly. Diagnosis can be made by molecular PCR, serologic testing such as anti-O157-antibody titer in serum, and confirmed by culture and strain isolation. Our patient was diagnosed by the stool molecular panel (PCR) which was positive for Shiga-toxin producing *E. coli*.

The mainstay of treatment is supportive with early hydration, blood transfusions if needed, and management of potential complications including acute kidney injury, malignant hypertension, and neurological sequelae. Complement inhibition by eculizumab the anti-C5 monoclonal antibody is not indicated but is sometimes used for severe neurological complications [9].

In fact, adequate hydration is at the core of HUS management. A meta-analysis in children with STEC-HUS published in 2017 showed that two predictors of poor prognosis are lack of early intravenous fluid administration and higher hematocrit on presentation [10]. Our patient received adequate IV hydration throughout his stay, and he had a good clinical response as well as improvement in his laboratory parameters. He was also transfused with one unit of 300 mL packed red blood cells (RBC).

Another important issue in the management of HUS is antibiotics. Their use is actually not recommended as they may increase the risk of HUS. A meta-analysis published in 2016 showed no overall association, but after excluding questionable studies, there was a significant association [11]. Our patient was not treated with antibiotics during his hospitalization. He gradually recovered after supportive therapy, as is the case for most patients with typical HUS. However, complications have been reported, and patients should be closely monitored and managed to reduce the risk of end-organ damage.

## Conclusions

Typical HUS in a previously healthy adult is a rare occurrence. In patients presenting with the classical triad of HUS in the right setting, a high index of suspicion is warranted. A thorough history and prompt stool samples are necessary to establish the diagnosis, and essentially rule out atypical HUS which has different management and poorer prognosis. Timely diagnosis of this rare syndrome allows adequate supportive management and decreases unnecessary laboratory tests and treatments such as antibiotics.
